# Supplementary material for: Generalism vs. specialization: Does niche breadth influence species responses to anthropogenic land-use change in Neotropical leaf-cutter ants?
Source: PLoS One. 2026 Oct 1;21(10):e0351518. doi: 10.1371/journal.pone.0351518 (PMC13630260; doi:10.1371/journal.pone.0351518)
Supplement: Supplementary file 2 [file pone.0351518.s001.docx]

**S1 Text. Disentangling the drivers of Principal Component 2 (PC2)**

To determine whether the high loadings of mound architecture (*Mound Area*, *Shape Ratio*) and *Soil pH* onto PC2 (Fig 2A; S6 Table) represented a causal ecosystem engineering mechanism (i.e., larger nests driving local soil modification), we constructed Generalized Linear Models (GLMs) using a Gaussian error distribution.

*Model 1. Additive effects model (without interaction):*

The first model evaluated the independent, direct effects of *Mound Area*, *Shape Ratio*, and *Species* identity on *Soil pH* under the formula:

Soil_pH ~ Species + Location + Mound_Area + Shape_Ratio

*Model 2. Conditional effects Model (with species interaction):*

To test the biological hypothesis that soil engineering capacity is species-specific (i.e., that the slope of *Soil pH* as a function of *Mound Area* differs between *A. cephalotes* and *A. laevigata*), we introduced a multiplicative interaction term under the formula:

Soil_pH ~ Mound_Area * Species + Shape_Ratio + Location

After accounting for sampling *Location*, the apparent relationship between colony traits and soil chemistry disappeared. Neither *Mound Area* (*P*= 0.641) nor *Shape Ratio* (*P* = 0.532) predicted *Soil pH* variation (S7 Table). Furthermore, the interaction between *Species* identity and *Mound Area* was non-significant (*P* = 0.136; S8 Table), demonstrating that this lack of architectural effect is consistent across both *Atta* species. Instead, the model's high explanatory power (Pseudo-*R*^2^ = 0.66) was driven entirely by spatial blocks (*Location* parameters such as *Location[T.Choro Bajo]*, *Location[T.El Raizal]*, *Location[T.Grima Baja]*, etc.).

We therefore conclude that the association between mound architecture and soil pH observed along PC2 does not reflect direct colony-mediated ecosystem engineering. Instead, it is more consistent with spatial differences among sampling locations, likely reflecting underlying edaphic conditions and land-use history.
