# Supplementary material for: Generalism vs. specialization: Does niche breadth influence species responses to anthropogenic land-use change in Neotropical leaf-cutter ants?
Source: PLoS One. 2026 Oct 1;21(10):e0351518. doi: 10.1371/journal.pone.0351518 (PMC13630260; doi:10.1371/journal.pone.0351518)
Supplement: Supplementary file 3 [file pone.0351518.s002.docx]

**S1 Table. Representative geographic coordinates and corresponding visualization codes for the study locations.**

| **Location** | **Two-letter code** | **Latitude (N)** | **Longitude (W)** |
| --- | --- | --- | --- |
| **Agua Buena** | AB | 6.28135 | -073.13047 |
| **Cantera** | CT | 6.17719 | -073.18733 |
| **Choro Bajo** | VN | 6.64588 | -073.16210 |
| **El Raizal** | RZ | 6.14935 | -073.18629 |
| **Grima Baja** | CB | 6.30970 | -073.15544 |
| **La Siberia** | LS | 6.27547 | -073.15166 |
| **La Victoria** | LV | 6.15613 | -073.18887 |
| **Montefrio** | MF | 6.27050 | -073.14447 |
| **Morario** | MO | 6.30960 | -073.13513 |
| **Nemizaque** | NZ | 6.22833 | -073.17700 |
| **Pozo Gallo** | PG | 6.26865 | -073.15557 |
| **Villas** | VI | 6.26149 | -073.16818 |

For each of the 12 sampled sites, a single representative GPS coordinate (decimal degrees, WGS 84 datum) is provided. The standardized two-letter abbreviation codes (Two-letter code) are explicitly defined here to facilitate spatial cross-referencing and improve graphical visualization in the box-plot distributions (S1 Fig).
