# Supplementary material for: Generalism vs. specialization: Does niche breadth influence species responses to anthropogenic land-use change in Neotropical leaf-cutter ants?
Source: PLoS One. 2026 Oct 1;21(10):e0351518. doi: 10.1371/journal.pone.0351518 (PMC13630260; doi:10.1371/journal.pone.0351518)
Supplement: Supplementary file 4 [file pone.0351518.s004.docx]

**S3 Table. Descriptive statistics and Shapiro–Wilk normality tests for ecological variables measured in *A. cephalotes* (C) and *A. laevigata* (L).**

| **Ecological Variable** | **Mean (C)** | **SD (C)** | **Mean (L)** | **SD (L)** | ***p*-norm (C)** | ***p*-norm (L)** | **Assumed Distribution** |
| --- | --- | --- | --- | --- | --- | --- | --- |
| **Mound area (m²)** | 45.015 | 37.452 | 69.059 | 61.899 | < 0.001 | < 0.001 | Non-normal |
| **Shape ratio** | 1.74 | 0.924 | 2.062 | 1.101 | < 0.001 | < 0.001 | Non-normal |
| **Soil pH** | 4.666 | 0.376 | 4.815 | 0.261 | 0.001 | 0.0008 | Non-normal |
| **Canopy openness (%)** | 67.668 | 18.493 | 89.57 | 6.919 | 0.0437 | < 0.001 | Non-normal |
| **Internal temperature ~10 cm (°C)** | 23.982 | 1.384 | 25.145 | 1.543 | 0.2191 | 0.0032 | Non-normal |
| **Thermal regulation capacity (°C)** | -7.07 | 4.388 | -10.32 | 4.7 | 0.2313 | 0.8717 | Normal |

Means, standard deviations (SD), and *p*-values are presented for each species. Variables were classified as normally distributed when both species showed *p* > 0.05. The departure from normality observed for internal temperature in *A. laevigata* is likely attributable to the truncated sampling period (ending at approximately 14:00), which captured only the warming phase of the daily thermal cycle.
