# Supplementary material for: Generalism vs. specialization: Does niche breadth influence species responses to anthropogenic land-use change in Neotropical leaf-cutter ants?
Source: PLoS One. 2026 Oct 1;21(10):e0351518. doi: 10.1371/journal.pone.0351518 (PMC13630260; doi:10.1371/journal.pone.0351518)
Supplement: Supplementary file 5 [file pone.0351518.s005.docx]

**S4 Table. Dispersion metrics for architectural and environmental variables.**

| **Variable** | **SD (C)** | **SD (L)** | **IQR (C)** | **IQR (L)** |
| --- | --- | --- | --- | --- |
| **Canopy openness (%)** | 18.493 | 6.755 | 30.325 | 4.987 |
| **Mound area (m²)** | 37.452 | 61.489 | 46.107 | 70.142 |
| **Shape ratio** | 0.924 | 1.08 | 0.74 | 0.933 |
| **Soil pH** | 0.376 | 0.261 | 0.6 | 0.3 |
| **Thermal regulation capacity (°C)** | 4.388 | 4.7 | 5.15 | 6.05 |

Intraspecific variation for *A. cephalotes* (C) and *A. laevigata* (L) is summarized using Standard Deviation (SD) and Interquartile Range (IQR). Larger values indicate greater within-species dispersion for the corresponding variable. Mound Area is reported in m² to facilitate ecological interpretation.
