# Supplementary material for: Generalism vs. specialization: Does niche breadth influence species responses to anthropogenic land-use change in Neotropical leaf-cutter ants?
Source: PLoS One. 2026 Oct 1;21(10):e0351518. doi: 10.1371/journal.pone.0351518 (PMC13630260; doi:10.1371/journal.pone.0351518)
Supplement: Supplementary file 6 [file pone.0351518.s006.docx]

**S5 Table. Percentage contribution of ecological and structural variables to the first two axes of the Principal Component Analysis (PCA).**

| **Variable** | **PC1 (%)** | **PC2 (%)** |
| --- | --- | --- |
| **Trees-type vegetation** | 28.4 | 0.14 |
| **Bush-type vegetation** | 25.95 | 0.06 |
| **Canopy openness (%)** | 23.7 | 1.3 |
| **Thermal regulation capacity (°C)** | 12.38 | 4.78 |
| **Mound area (m²)** | 5.34 | 30.18 |
| **Grass-type vegetation** | 3.33 | 5.66 |
| **Shape ratio** | 0.47 | 27.8 |
| **Soil pH** | 0.43 | 30.08 |

Variables are ordered by their contribution to PC1. High contributions on PC1 reflect local vegetation density and canopy architecture, whereas high loadings on PC2 differentiate colony dimensions (mound area and shape ratio) and chemical soil properties (pH).
