# Supplementary material for: Generalism vs. specialization: Does niche breadth influence species responses to anthropogenic land-use change in Neotropical leaf-cutter ants?
Source: PLoS One. 2026 Oct 1;21(10):e0351518. doi: 10.1371/journal.pone.0351518 (PMC13630260; doi:10.1371/journal.pone.0351518)
Supplement: Supplementary file 7 [file pone.0351518.s007.docx]

**S6 Table. Indicator Value (IndVal) analysis of vegetation categories (Grasses, Bushes, and Trees) within a 10 m radius of *A. cephalotes* and *A. laevigata mounds*.**

| **Species** | **Vegetation Category** | **Specificity (A)** | **Fidelity (B)** | **IndVal Index** |
| --- | --- | --- | --- | --- |
| ***A. cephalotes*** | Bushes | 0.783 | 0.964 | 0.869 |
| ***A. cephalotes*** | Trees | 0.785 | 0.911 | 0.845 |
| ***A. cephalotes*** | Grasses | 0.482 | 0.946 | 0.675 |
| ***A. laevigata*** | Grasses | 0.518 | 0.983 | 0.714 |
| ***A. laevigata*** | Bushes | 0.217 | 0.259 | 0.237 |
| ***A. laevigata*** | Trees | 0.215 | 0.241 | 0.228 |

Because vegetation categories were not mutually exclusive, indices were calculated based on the independent presence of each structural component. Specificity (A) indicates the exclusivity of a species to a vegetation category, Fidelity (B) indicates the frequency of the category's occurrence with the species, and the IndVal index represents the combined indicative strength.
