# Supplementary material for: Generalism vs. specialization: Does niche breadth influence species responses to anthropogenic land-use change in Neotropical leaf-cutter ants?
Source: PLoS One. 2026 Oct 1;21(10):e0351518. doi: 10.1371/journal.pone.0351518 (PMC13630260; doi:10.1371/journal.pone.0351518)
Supplement: Supplementary file 8 [file pone.0351518.s008.docx]

**S7 Table. Generalized Linear Model (GLM) regression results for the additive effects of colony architecture and species identity on soil pH.**

|  | **Coef.** | **Std.Err.** | ***z*** | ***p* > \|*z*\|** | **[0.025** | **0.975]** |
| --- | --- | --- | --- | --- | --- | --- |
| **Intercept** | 4.6012 | 0.115 | 40.021 | 0 | 4.3759 | 4.8265 |
| **Species[T.L]** | -0.0118 | 0.0669 | -0.177 | 0.8597 | -0.1429 | 0.1193 |
| **Location[T.Cantera]** | -0.2107 | 0.156 | -1.351 | 0.1768 | -0.5165 | 0.095 |
| **Location[T.Choro Bajo]** | 0.3801 | 0.0986 | 3.856 | 0.0001 | 0.1869 | 0.5733 |
| **Location[T.El Raizal]** | -0.6155 | 0.1416 | -4.347 | 0 | -0.893 | -0.338 |
| **Location[T.Grima Baja]** | 0.323 | 0.1047 | 3.086 | 0.002 | 0.1178 | 0.5281 |
| **Location[T.La Siberia]** | 0.2149 | 0.1046 | 2.055 | 0.0399 | 0.0099 | 0.4199 |
| **Location[T.La Victoria]** | -0.2022 | 0.1296 | -1.56 | 0.1187 | -0.4563 | 0.0518 |
| **Location[T.Montefrio]** | -0.0161 | 0.1065 | -0.152 | 0.8795 | -0.2248 | 0.1925 |
| **Location[T.Morario]** | 0.3478 | 0.1445 | 2.407 | 0.0161 | 0.0646 | 0.6311 |
| **Location[T.Nemizaque]** | 0.234 | 0.1017 | 2.302 | 0.0213 | 0.0348 | 0.4333 |
| **Location[T.Pozo Gallo]** | 0.2895 | 0.1239 | 2.337 | 0.0194 | 0.0467 | 0.5324 |
| **Location[T.Villas]** | 0.3018 | 0.1635 | 1.846 | 0.0649 | -0.0186 | 0.6223 |
| **Mound_Area** | 0 | 0 | -0.467 | 0.6405 | 0 | 0 |
| **Shape_Ratio** | 0.0149 | 0.0239 | 0.625 | 0.532 | -0.0319 | 0.0617 |
| Model Fit: No. Obs = 111 \| Df Residuals = 96 \| Pseudo R-squ. (CS) = 0.6501 \| Deviance = 5.4869 | | | | | | |

The model was fitted using a Gaussian error distribution and an identity link function (*N* = 111 colonies, reflecting the subset of the 114 total downstream colonies with complete soil pH records). Geographic localities were incorporated as categorical dummy variables to serve as spatial blocks, isolating landscape-level edaphic baselines. The reference baseline (Intercept) reflects a baseline geographic control site. Coefficients (β), standard errors (SE), Wald *z*-scores, and corresponding *p*-values are provided for each predictor variable.
