# Supplementary material for: Generalism vs. specialization: Does niche breadth influence species responses to anthropogenic land-use change in Neotropical leaf-cutter ants?
Source: PLoS One. 2026 Oct 1;21(10):e0351518. doi: 10.1371/journal.pone.0351518 (PMC13630260; doi:10.1371/journal.pone.0351518)
Supplement: Supplementary file 9 [file pone.0351518.s009.docx]

**S8 Table. Generalized Linear Model (GLM) regression results evaluating the conditional effects and species-specific interactions of colony traits on soil pH.**

|  | **Coef.** | **Std.Err.** | ***z*** | ***p* > \|*z*\|** | **[0.025** | **0.975]** |
| --- | --- | --- | --- | --- | --- | --- |
| **Intercept** | 4.6301 | 0.1159 | 39.954 | 0 | 4.403 | 4.8572 |
| **Species[T.L]** | -0.1075 | 0.0924 | -1.163 | 0.245 | -0.2886 | 0.0737 |
| **Location[T.Cantera]** | -0.2146 | 0.1551 | -1.384 | 0.1664 | -0.5185 | 0.0893 |
| **Location[T.Choro Bajo]** | 0.4232 | 0.1022 | 4.143 | 0 | 0.223 | 0.6235 |
| **Location[T.El Raizal]** | -0.588 | 0.1419 | -4.143 | 0 | -0.8661 | -0.3098 |
| **Location[T.Grima Baja]** | 0.3565 | 0.1064 | 3.35 | 0.0008 | 0.1479 | 0.565 |
| **Location[T.La Siberia]** | 0.2436 | 0.1057 | 2.305 | 0.0212 | 0.0364 | 0.4508 |
| **Location[T.La Victoria]** | -0.1997 | 0.1288 | -1.55 | 0.1211 | -0.4522 | 0.0528 |
| **Location[T.Montefrio]** | -0.0081 | 0.1059 | -0.077 | 0.9389 | -0.2158 | 0.1995 |
| **Location[T.Morario]** | 0.3569 | 0.1437 | 2.483 | 0.013 | 0.0752 | 0.6387 |
| **Location[T.Nemizaque]** | 0.2803 | 0.1057 | 2.652 | 0.008 | 0.0731 | 0.4874 |
| **Location[T.Pozo Gallo]** | 0.3332 | 0.1266 | 2.633 | 0.0085 | 0.0852 | 0.5813 |
| **Location[T.Villas]** | 0.324 | 0.1632 | 1.986 | 0.0471 | 0.0042 | 0.6438 |
| **Mound_Area** | 0 | 0 | -1.502 | 0.1332 | 0 | 0 |
| **Mound_Area:Species[T.L]** | 0 | 0 | 1.489 | 0.1364 | 0 | 0 |
| **Shape_Ratio** | 0.0162 | 0.0237 | 0.682 | 0.4951 | -0.0303 | 0.0627 |
| Model Fit: No. Obs = 111 \| Df Residuals = 95 \| Pseudo R-squ. (CS) = 0.6620 \| Deviance = 5.3617 | | | | | | |

The model utilizes a Gaussian error distribution and an identity link function (*N* = 111 colonies, reflecting the subset of the 114 total downstream colonies with complete soil pH records) to test the biological hypothesis that soil engineering capacity varies as a function of species identity (represented by the multiplicative interaction term Mound_Area:Species[T.L]). Geographic localities are controlled via spatial blocking parameters. Coefficients (β), standard errors (SE), Wald *z*-scores, and *p*-values are reported for all fixed effects and spatial block contrasts.
