# Supplementary material for: Generalism vs. specialization: Does niche breadth influence species responses to anthropogenic land-use change in Neotropical leaf-cutter ants?
Source: PLoS One. 2026 Oct 1;21(10):e0351518. doi: 10.1371/journal.pone.0351518 (PMC13630260; doi:10.1371/journal.pone.0351518)
Supplement: Supplementary file 10 [file pone.0351518.s010.docx]

**S1 Fig. Multivariate assessment of mound architecture, edaphic properties, and thermal regulation across sampling locations.** Box-and-whisker plots **(A–E)** illustrate the intraspecific and interspecific variation for A. *cephalotes* (green) and *A. laevigata* (orange) across twelve distinct geographic sites. **(A)** *Mound Area*, presented as Log_10_-transformed values (cm^2^) to normalize the highly skewed distribution of colony sizes. **(B)** *Shape Ratio*, defined as the degree of surface symmetry. **(C)** **Soil pH**, representing the chemical conditions of the mound substrate. **(D)** *Light Percentage*, indicating the level of canopy openness at the nest site. **(E)** *Temperature Difference*, representing the thermal gradient between the internal colony core at ~10 cm and the external environment (°C). Shaded boxes denote the interquartile range (IQR), horizontal lines represent the median, and whiskers extend to 1.5 times the IQR. Two-letter codes at the base of each distribution correspond to specific sampling locations (e.g., MF: Montefrio, NZ: Nemizaque, etc.; see S1 Table for full location metadata).
