# Supplementary material for: Generalism vs. specialization: Does niche breadth influence species responses to anthropogenic land-use change in Neotropical leaf-cutter ants?
Source: PLoS One. 2026 Oct 1;21(10):e0351518. doi: 10.1371/journal.pone.0351518 (PMC13630260; doi:10.1371/journal.pone.0351518)
Supplement: Supplementary file 11 [file pone.0351518.s011.docx]

**S2 Fig. Diel thermal profiles of *Atta* colony interiors.** Points represent individual temperature measurements (n = 114) recorded at approximately 10 cm depth for *A. cephalotes* (green circles) and *A. laevigata* (orange crosses). Trend lines represent a second-order polynomial regression (*R*^2^ fit), with shaded ribbons indicating the 95% confidence intervals. *A. laevigata* colonies exhibited significantly higher internal temperatures compared to *A. cephalotes* (PERMANOVA test: ΔT = 1.2°C, *p* = 0.001; S3 and S4 Tables). The widening of the confidence interval for *A. laevigata* after 14:00 reflects the increased statistical uncertainty at the upper bound of the sampling window for this species.

**S3 Fig. Regional average February temperature across the study area in Santander, Colombia, derived from ERA5-Land reanalysis data.** Panels **(A–C)** show the average 2 m air temperature (°C) for February 2024, 2025, and 2026, respectively, using a shared colour scale ranging from 16.74°C (yellow; lower temperatures) to 22.92°C (deep red; higher temperatures). All climatic heatmaps are georeferenced and overlaid on an OpenStreetMap basemap of the sampling region. Black points indicate the fieldwork locations where *Atta* colonies were surveyed, with location names provided for geographic reference across the latitudinal and elevational gradient of the study area. Base map and data from OpenStreetMap and OpenStreetMap Foundation. The map contains information from OpenStreetMap and OpenStreetMap Foundation, which is made available under the Open Database License. Map generated in Python 3.12.3 using Matplotlib, GeoPandas, Contextily, xarray, and rioxarray.

**S4 Fig. Regional total precipitation across the study area in Santander, Colombia, derived from ERA5-Land reanalysis data.** Panels **(A–B)** show the total annual accumulated precipitation (m) in 2024 and 2025, respectively, using a shared colour scale ranging from approximately 1.6 m (yellow; lower precipitation) to 2.5 m (deep blue; higher precipitation). Panel **(C)** shows cumulative precipitation from January to July 2026 and uses a separate colour scale because it represents a partial year. All climatic heatmaps are georeferenced and overlaid on an OpenStreetMap basemap of the sampling region. Black points indicate the fieldwork locations where *Atta* colonies were surveyed, with location names provided for geographic reference across the latitudinal and elevational gradient of the study area. Base map and data from OpenStreetMap and OpenStreetMap Foundation. The map contains information from OpenStreetMap and OpenStreetMap Foundation, which is made available under the Open Database License. Map generated in Python 3.12.3 using Matplotlib, GeoPandas, Contextily, xarray, and rioxarray.

**S5 Fig. Regional total bare-soil evaporation across the study area in Santander, Colombia, derived from ERA5-Land reanalysis data.** Panels **(A–B)** show the total annual accumulated bare-soil evaporation (mm) in 2024 and 2025, respectively, using a shared colour scale ranging from approximately 267 mm (yellow; lower evaporation) to 377 mm (deep blue; higher evaporation). Panel **(C)** shows cumulative bare-soil evaporation from January to July 2026 and uses a separate colour scale because it represents a partial year. All climatic heatmaps are georeferenced and overlaid on an OpenStreetMap basemap of the sampling region. Black points indicate the fieldwork locations where *Atta* colonies were surveyed, with location names provided for geographic reference across the latitudinal and elevational gradient of the study area. Base map and data from OpenStreetMap and OpenStreetMap Foundation. The map contains information from OpenStreetMap and OpenStreetMap Foundation, which is made available under the Open Database License. Map generated in Python 3.12.3 using Matplotlib, GeoPandas, Contextily, xarray, and rioxarray.

## **Supporting datasets**

**S1 Dataset. Characterization of canopy openness across sampled colonies via hemispherical photography.** This PDF document compile the complete photographic registry for all 117 surveyed *Atta* colonies along the regional transect. Each row presents a pairwise comparison containing: (left) the raw hemispherical photograph converted to greyscale, and (right) the binarized silhouette version resulting from the interactive threshold masking process. Each pair is labelled with its unique image identifier (Photo_File), the selected binarization algorithm context (Sky: S [sunny], C [cloudy] or P [partially cloudy]), the specific mathematically applied Gamma correction factor, and the final estimated light percentage calculated over the mound. This light percentage serves as our proxy for canopy openness used in all downstream niche breadth and partitioning multivariate analyses.

**S2 Dataset. Ground-level photographic registry of colony mound architectures.** This compressed file (Supplementary_Dataset_S2.zip) contains the complete repository of fieldwork photographs of mounds for the 117 surveyed *Atta* colonies. The dataset includes wide-angle ground-level photographs of each distinct nest mound, captured utilizing the same hemispherical fish-eye lens system described for the canopy sub-canopy characterization in the *Materials and Methods* section. Additionally, some standard-format (non-distorted) landscape photographs are provided. While these visual records were not deployed as quantitative variables in downstream statistical modelling, they are archived here to provide transparent empirical evidence and an environmental record of the regional fieldwork. The .zip file was deposited at Zenodo and it is publicly available at <https://doi.org/10.5281/zenodo.20342937>
