# Supplementary material for: Generalism vs. specialization: Does niche breadth influence species responses to anthropogenic land-use change in Neotropical leaf-cutter ants?
Source: PLoS One. 2026 Oct 1;21(10):e0351518. doi: 10.1371/journal.pone.0351518 (PMC13630260; doi:10.1371/journal.pone.0351518)
Supplement: Supplementary file 14 [file pone.0351518.s014.docx]

**S5 Fig. Regional total bare-soil evaporation across the study area in Santander, Colombia, derived from ERA5-Land reanalysis data.** Panels **(A–B)** show the total annual accumulated bare-soil evaporation (mm) in 2024 and 2025, respectively, using a shared colour scale ranging from approximately 267 mm (yellow; lower evaporation) to 377 mm (deep blue; higher evaporation). Panel **(C)** shows cumulative bare-soil evaporation from January to July 2026 and uses a separate colour scale because it represents a partial year. All climatic heatmaps are georeferenced and overlaid on an OpenStreetMap basemap of the sampling region. Black points indicate the fieldwork locations where *Atta* colonies were surveyed, with location names provided for geographic reference across the latitudinal and elevational gradient of the study area. Base map and data from OpenStreetMap and OpenStreetMap Foundation. The map contains information from OpenStreetMap and OpenStreetMap Foundation, which is made available under the Open Database License. Map generated in Python 3.12.3 using Matplotlib, GeoPandas, Contextily, xarray, and rioxarray.
