# Supplementary material for: Generalism vs. specialization: Does niche breadth influence species responses to anthropogenic land-use change in Neotropical leaf-cutter ants?
Source: PLoS One. 2026 Oct 1;21(10):e0351518. doi: 10.1371/journal.pone.0351518 (PMC13630260; doi:10.1371/journal.pone.0351518)
Supplement: Supplementary file 15 [file pone.0351518.s016.docx]

**S2 Dataset. Ground-level photographic registry of colony mound architectures.** This compressed file (Supplementary_Dataset_S2.zip) contains the complete repository of fieldwork photographs of mounds for the 117 surveyed *Atta* colonies. The dataset includes wide-angle ground-level photographs of each distinct nest mound, captured utilizing the same hemispherical fish-eye lens system described for the canopy sub-canopy characterization in the *Materials and Methods* section. Additionally, some standard-format (non-distorted) landscape photographs are provided. While these visual records were not deployed as quantitative variables in downstream statistical modelling, they are archived here to provide transparent empirical evidence and an environmental record of the regional fieldwork. The .zip file was deposited at Zenodo and it is publicly available at <https://doi.org/10.5281/zenodo.20342937>
