# Supplementary material for: Generalism vs. specialization: Does niche breadth influence species responses to anthropogenic land-use change in Neotropical leaf-cutter ants?
Source: PLoS One. 2026 Oct 1;21(10):e0351518. doi: 10.1371/journal.pone.0351518 (PMC13630260; doi:10.1371/journal.pone.0351518)
Supplement: Supplementary file 16 [file pone.0351518.s017.docx]

# **Response to Reviewers**

Dear Academic Editor,

Please find below the responses to each point of the first revision round of our manuscript, "**Generalism vs. specialization: Does niche breadth influence species responses to anthropogenic land-use change in Neotropical leaf-cutter ants?**" (Manuscript ID: **PONE-D-26-26201**). We found the comments from the Editor and Reviewers extremely helpful in improving the quality of our manuscript, and we would like to take this opportunity to express our sincere gratitude.

## **Summary of changes**

First, after considering the reviewers’ comments, we revised the title of the manuscript to better reflect the scope and main focus of the study.

**Former title:** *Does niche breadth predict resilience to anthropogenic land-use change in ecosystem engineers?*

**New title:** *Generalism vs. specialization: Does niche breadth influence species responses to anthropogenic land-use change in Neotropical leaf-cutter ants?*

One major point that required attention was the conceptual framework of the study, which in the previous version relied on plasticity versus specialization. Following the valuable comments from the Editor and Reviewer 2, we carefully reconsidered this framework and concluded that the concept of plasticity did not accurately reflect either the purpose of the study or the evidence generated by our analyses. We therefore adopted a generalist versus specialist framework. Accordingly, we reformulated our hypotheses and expanded the literature review to better represent the current state of research on niche breadth, also addressing the Editor's concern regarding the limited coverage of recent literature. This revision also resolved Reviewer 3's concern regarding the former Plastic Survivor versus Specialist Filter hypotheses.

As correctly pointed out by the Editor, in the former version of the manuscript many of the principal statistical results were embedded in the text, while the corresponding table was placed in the Supplementary Information. We therefore moved the former S4 Table to the main manuscript as Table 1 and refined the Results section to improve clarity and avoid unnecessary repetition. Likewise, we carefully revised the manuscript to ensure that results were presented only in the Results section and that interpretation remained within the Discussion.

In S3 Fig, we added four additional panels showing total annual precipitation (m) and total annual evaporation (mm) for both 2024 and 2026. This addition addresses Reviewer 1's concern regarding the consistency and comparability of the climatic data presented in the manuscript.

Reviewer 3 suggested expanding the Discussion to consider the potential role of predation. We therefore incorporated a dedicated paragraph discussing the natural predators of *Atta* species described in the literature, as well as the traditional harvesting of *A. laevigata* for human consumption in the Santander region of Colombia. We emphasize that the potential contribution of predation and harvesting to the contrasting responses of both species to land-use change remains unknown and deserves further investigation.

In addition to substantially expanding the literature review with recent publications (2020–2026) on niche breadth, anthropogenic environmental filters, and land-use change, we also incorporated appropriate citations for the Python libraries and statistical approaches used throughout the study, following the Editor's recommendations.

The Editor also emphasized the need to substantially rewrite the manuscript to minimize AI-generated text. We carefully revised the entire manuscript and extensively rewrote numerous sections. We sincerely apologize for our oversight in the original submission. We also clarified the way AI-assisted tools were used and incorporated a dedicated AI Declaration within the Materials and Methods section.

The editorial office also requested clarification regarding permissions for access to the sampling sites. Accordingly, we added a dedicated statement to the Materials and Methods (Lines 176–185). Most sampling was conducted in publicly accessible areas intersected by public roads and footpaths, where no access permits were required. For colonies located on privately owned properties (including Choro Bajo, Morario, Grima Baja, El Raizal, and Montefrío), permission to access the sites was obtained from the respective landowners or property managers, several of whom accompanied the fieldwork. Because no biological specimens were collected for scientific purposes and all ecological measurements were performed in situ, no scientific collecting permit was required.

**Figure 1A–B:** To address the copyright and image-source clarification, panels A and B of Figure 1 have been replaced with cropped versions of the original photographs taken by the author. The original millimeter-grid paper background is now visible in both panels. Only cropping was applied for composition; no other image editing or processing was performed. The author is the original copyright holder of these images. We deposited the original image files (.jpg) in the GitHub repository of the project, associated to a Zenodo DOI, which is described in the “Data availability” statement..

We deposited the complete source code used in this study in Zenodo, together with the ground-level photographs of the nests and the hemispherical photographs used for canopy analyses. Consequently, we added a Data Availability Statement (Line 705).

Finally, we addressed the Editor's request regarding permission to use the OpenTopoMap layers previously displayed in Fig 1 and S3 Fig, as well as in the newly added S4 and S5 Figs. We removed the OpenTopoMap relief layer and replaced it with an OpenStreetMap basemap, which is distributed under a license compatible with PLOS ONE's requirements. The corresponding attribution to OpenStreetMap contributors is now provided in the figure legends.

**Author affiliation:** Since the initial submission, the author affiliation has been updated from “Independent Researcher” to “Institute of Science and Technology Austria (ISTA), Klosterneuburg, Austria.” Although this study was conducted independently and outside the scope of my former employment at ISTA, the Institute has explicitly authorized the use of the ISTA affiliation for this publication.

## **Editor**

This paper shows clear evidence of extensive use of artificial intelligence in most (if not all) sections and steps of the write-up. Yet there is no indication of anywhere that the authors have made any declarations of the same. Use of AI in any aspect of the paper must be explicitly declared, with details of what sections and procedures it was subjected to, and which ones in in which it was not. Otherwise the presentation is considered plagiarized as it was not the work of the authors and therefore will have to be rejected without any further consideration.

Thanks for pointing out the missing AI-declaration statement. We added in the Materials and methods section a new subsection called *Use of AI-assisted technologies* (Line 328) where we explicitly declare and describe the use of AI tools during manuscript preparation. The revised manuscript now specifies that we used AI exclusively for language editing and limited programming assistance, while all hypotheses, study design, field data collection, statistical analyses, interpretations, and conclusions were developed and verified entirely by the authors.

In revising the article, the authors should, in addition to their declaration on AI use, re-write the paper in a structure and format that strictly complies with the journal’s guidelines, ensuring that AI use does not constitute or contribute to more than 30% of actual human input. The journal has elaborate mechanisms for detecting these contributions.

We appreciate the Editor’s concern regarding the use of AI-assisted writing. In addition to including an explicit AI-use declaration in the Materials and Methods section (Line 328), we thoroughly revised the entire manuscript to ensure full compliance with the journal’s guidelines. We critically re-examined all sections of the manuscript and rewrote substantial part of the text where necessary to ensure that the final wording accurately reflects our original scientific ideas, interpretations, and intended meaning.

The reviewers laud the importance of the research topic and general structuring of the research, the framing of the research questions and overall analytical framework, although one reviewer was particularly concerned about certain aspects of interpretation of the results, For instance, the authors need to clearly justify in Introduction section why plasticity and specialization were considered as mutually exclusive and opposite strategies by the two ant species/groups, especially given that specialist may at times also adopt plasticity tendencies depending on prevailing environmental/habitat conditions. In particular, a robust theoretical framework must be provided in the literature review (Introduction) and in the Methods to demonstrate the extent to which plasticity and specialization may be regarded as being mutually exclusive and opposite to each other with regards to the current study scenario and the taxa involved. If there is no justification for such exclusivity, consider using the term ‘generalist’ instead of ‘plasticity’, which should therefore be toned down throughout the Discussion and conclusion.

We thank the Editor and Reviewer 2 for this important conceptual observation. We carefully reconsidered the theoretical framework of our study and agree that the term plasticity did not accurately reflect either our hypotheses or the evidence generated by our analyses. Accordingly, we substantially revised the manuscript by **replacing the former plasticity versus specialization framework** with a more appropriate **generalist versus specialist framework**, which is better supported by the ecological literature on niche breadth.

Throughout the manuscript (e.g. Lines 25, 64–66, 487–491), we now interpret the broader realized niche of *A. cephalotes* as being consistent with a more generalist ecological strategy, whereas the narrower realized niche of *A. laevigata* is discussed as being consistent with a more specialist strategy. We also moderated our interpretations and conclusions by emphasizing that our analyses quantify only realized niche breadth across the environmental variables included in this study, rather than directly demonstrating generalist or specialist strategies.

Can the authors justify why many elements of Results (including Figure/Figure captions) are presented in Methods?

We thank the Editor for this important observation. We carefully restructured the manuscript to ensure a clear separation between the Introduction, Materials and Methods, Results, and Discussion, following the journal's recommendations. In the revised version, methodological descriptions are now strictly limited to the procedures used in the study. All observations, statistical outcomes, and interpretations were moved to the Results and Discussion sections. We also revised all figure titles and captions to ensure that they are descriptive rather than interpretative.

Specifically, we implemented the following changes:

*Introduction*

We removed from the paragraph Lines 124-141 the part were we explicitly had said that mound architecture was “a proxy for behavioral plasticity”.

The hypotheses were reformulated to avoid implying expected results (e.g., replacing the previous "Plastic Survivor" and "Specialist Filter"). Instead of assuming that *A. cephalotes* possesses a broader niche, we now explicitly state that we tested whether differences in realized niche breadth are associated with contrasting persistence in human-modified landscapes (Lines 135-138).

*Materials and Methods*

Statements implying causal outcomes (e.g. "ensuring the strict independence..." of the colonies ) were replaced by more appropriate methodological wording ("minimized the likelihood..." for territorial overlapping ) in line 158.

We expanded descriptions of statistical analyses (Lines 249-271) to explain the analytical workflow (distance matrices, standardization, permutations, PERMDISP, and *R*^2^ effect size calculations).

The regional climate section was revised to remove interpretative statements such as correlating colony occurrence with climatic gradients (Lines 323-327). This way, we limited this section to process and visualize ERA5-Land data.

*Results*

The Results section was reorganized into thematic subsections with descriptive titles reflecting the principal findings.

*Figures and figure captions*

Figure titles were rewritten to be descriptive (e.g. *Fig 2. Fine-scale realized niche characterization and habitat affinity* instead of *Fig 2. Multivariate niche partitioning and habitat affinity*).

Figure captions were revised to describe only what is displayed, removing interpretative statements that could be considered part of the Results or Discussion. In particular, in Fig 2, we removed the expression “Note the significant niche contraction in canopy openness and soil pH for *A. laevigata* contrasted with the architectural specialization (compact mound area) of *A. cephalotes*”.

*Discussion*

We expanded the Discussion with recent literature on realized niche breadth and land-use change across multiple taxa, allowing our findings to be interpreted within a broader ecological context (Lines 584-590).

There is almost no literature cited to accompany the analysis steps outlined in Methods. To ensure reproducibility, which is a requirement for all research, each analysis step must be supported with details of corresponding analysis tool used, software version and cited literature, unless the techniques are the authors’ original ideas.

We expanded the Materials and Methods to improve the reproducibility of all analytical procedures as requested. In the revised manuscript, each analytical step is now accompanied by a detailed description of the workflow, the corresponding software or Python library (including version numbers), and the relevant methodological references whenever applicable.

Specifically, we implemented the following changes:

We now report the versions of all principal Python libraries used in the analyses, including scikit-learn, SciPy, scikit-bio, and cdsapi, together with the corresponding citations.

The statistical analyses were expanded to describe the analytical workflow. For example, we now specify the use of *z*-score standardization, the construction of Euclidean distance matrices of dissimilarity, the implementation of PERMANOVA and PERMDISP, the number of permutations (999), grouping variables, and software implementations used in each analysis (Lines 249-271).

We incorporated the original methodological references supporting the statistical procedures, including Anderson (2001) for PERMANOVA and Anderson (2006) for PERMDISP.

Because the Python implementation of PERMANOVA does not report effect sizes, we explicitly describe how we derived *R*² values algebraically from the pseudo-*F* statistic, including the corresponding equation and justification for its use.

For the PCA (Lines 272-282) and Indicator Value (IndVal; Lines 283-290) we also included the software libraries that we used as well as the statistical frameworks: PCA according to Jolliffe (2002) and IndVal following Dufrêne & Legendre (1997) and De Cáceres & Legendre (2009).

The authors need to present (directly in the text body rather than supplementary material), many more results in terms of illustration such as tables and graphs/plots, especially from analyses involving PERMANOVA. PERMANOVA results are best presented in full within a table rather than in-text, including details of the F statistic, effect sizes, permutation p, the actual p and degrees of freedom plus the relative percent contributions of each predictor variable.

We thank the Editor for this valuable suggestion. We revised the presentation of the multivariate statistical results to improve their accessibility and comply with the journal's recommendations as follows:

The complete PERMANOVA and PERMDISP results, previously presented only in the Supplementary Information (former S4 Table), were moved to the main manuscript and are now presented as Table 1 (Line 369).

Table 1 now reports the principal PERMANOVA and PERMDISP results for each environmental variable, including species means, standard deviations, pseudo-*F* statistics, ***R*² effect sizes**, permutation-based *p*-values, and PERMDISP significance tests. Because all analyses consisted of independent one-factor PERMANOVAs comparing the same two species (N = 114), the numerator and denominator **degrees of freedom** were identical across variables and therefore were not repeated in the table.

The **relative contributions of individual environmental variables** to multivariate niche differentiation are presented separately through the PCA loading vectors and eigenvector analysis (Fig. 2A; S5 Table), which complement the PERMANOVA results by identifying the principal environmental gradients underlying species separation.

Following this reorganization, all elements of the Supplementary Information were renumbered accordingly throughout the manuscript.

Because the complete statistical results are now available in Table 1, we revised the Results subsection "Realized niche characterization reveals fine-scale ecological differences between sympatric Atta species" to avoid unnecessary repetition of the numerical values already presented in the table. The text now focuses on describing the principal ecological patterns, while Table 1 provides the detailed statistical outputs.

These modifications improve the presentation of the multivariate analyses and make the principal statistical results immediately available in the main text, as recommended.

There is need for a small separate Conclusion section summarizing the extent to which the hypotheses were proved or otherwise.

Following the Editor's recommendation, we added a separate Conclusion section at the end of the main text (Line 688).

In terms of cited literature, the depth of literature review appears a bit shallow for a research theme (niche breadth assessment) which has seen very vigorous research across the world over the past 6 years. With a total of only 51 references, and majority of them more than 15 years old, the authors need to review more recent literature to provide a more robust case for their contribution to the subject, and to more clearly justify a research gap and novelty.

We thank the Editor for highlighting this opportunity to strengthen the literature review. We expanded both the Introduction and Discussion by incorporating more than a dozen recent publications (2020–2026) addressing niche breadth, ecological specialization, environmental filtering, and species persistence under land-use change. With these additions we strengthen the theoretical framework, better justify the research gap, and place our findings within the context of recent advances in the field.

Specifically, we incorporated studies on the role of land-use change as an ecological filter (e.g. Boyle et al., 2021; Winkler et al., 2021; Lines 41-45) and the contrasting responses of ecological "winners" and "losers" in this context (Filgueiras et al., 2021; Lines 48-51, 67-70). We also updated the conceptual framework of niche breadth together with modern perspectives on specialization and niche dimensionality (Carscadden et al., 2020; Di Cecco & Hurlbert, 2022; Lines 61-70).

To better support the relationship between niche breadth and species persistence, we added recent empirical studies demonstrating that species with narrower niches are generally more vulnerable to land-use change (Wehner et al., 2021; Lines 67-70). We further incorporated studies and reviews referring to specific taxa, including butterflies (Riva et al., 2023; Lines 76-79, 584-587), birds (Liordos et al., 2021; Avidad et al., 2025; Lines 72-74), insects (Waldock et al., 2020; Lines 74-76), and amphibians (Kearney et al., 2025; Lines 74-76), which collectively show that broader environmental niches are associated with greater persistence in human-modified landscapes.

These additions also helped us refine the research gap. In Lines 79-85 and 584-590 we now explain that many previous studies infer niche breadth from broad geographic distributions, species occurrence records, or coarse habitat categories rather than from direct measurements of fine-scale environmental conditions. Therefore, our research complement previous large-scale studies by demonstrating that fine-scale measurements of realized niche breadth are likewise consistent with the greater persistence of species exhibiting broader realized niches in human-modified landscapes.

The reference list increased from 51 to 82 citations (including citations to the main python libraries and statistical approaches), with the majority of the newly incorporated references in the context of niche-breadth and land-use change published between 2020 and 2026.

There are many more specific issues raised by the reviewers and the authors need to address each of these point by point in their Major revision, and submit a separate account of how this has been accomplished, besides a clean and a track-changed version of the manuscript.

**Reviewer 1**

Thank you to the author for this interesting manuscript and to the Editor for inviting me to review it. I am not an animal ecologist by training, but I accepted this review out of curiosity, to see whether responses to environmental characteristics and resource-use strategies are similar to those observed in the plant world. The manuscript is very well structured and clearly written. It is easy to read, and the key aspects of the study system, methods, analyses, and results are presented in a clear and understandable manner.

We sincerely thank the Reviewer for the careful review of our manuscript and for the positive assessment of its structure, clarity, and readability. We are grateful for the constructive comments and suggestions, which have helped us strengthen both the conceptual framework and the presentation of our study.

A few minor comments and suggestions:

Line 107: replace “(Cherrett, 1968; Wirth et al., 2007)” with “[33, 36]”.

Thanks for the observation, we corrected such inconsistencies in the citation style (Line 96).

Lines 161–162: I would suggest a slight rephrasing. Instead of: “Specifically, ten locations were sampled in the rural area of the municipality of Charalá, one in Ocamonte, and one in Barichara (S1 Table, Fig 1C).” perhaps: “Specifically, 12 locations were sampled: 10 in the rural area of the municipality of Charalá, 1 in Ocamonte, and 1 in Barichara (S1 Table, Fig 1C).”

We agreed with the proposed rephrasing (Lines 147-149).

Line 176: where it states “Only active colonies were included in the study,” as a reader I initially expected that all colonies would have been surveyed. Including inactive nests could potentially have strengthened the hypothesis that one species is more sensitive than the other to certain environmental changes. However, I realize that abandoned nests may be difficult to locate and identify reliably.

We thank the Reviewer for this thoughtful observation. As correctly inferred, we deliberately restricted our sampling to active colonies because species identification based on nest characteristics alone would be unreliable once colonies have been abandoned. In addition, colony inactivity may result from multiple causes unrelated to natural environmental conditions, including disease, colony aging, or human intervention (e.g., insecticide application), making the ecological interpretation of abandoned nests difficult. Nevertheless, we agree with the Reviewer that incorporating inactive colonies into future studies, where the causes of colony abandonment can be reliably determined, could provide valuable insights into *Atta* persistence under land-use change.

One final question concerns the climatic data. Temperature data were collected for the month of February across three years (2024, 2025, and 2026), whereas precipitation data were considered only for 2025, covering all months of the year. This does not affect the readability of the manuscript, and I do not believe it is likely to alter the conclusions substantially. Nevertheless, the question naturally arises from the standpoint of data consistency and comparability.

We thank the Reviewer for this helpful observation. To improve data consistency and comparability across years, we expanded the former S3 Fig into three separate figures (S3–S5 Figs), each dedicated to a different climatic variable. S3 Fig now presents the average February temperature for 2024, 2025, and 2026. S4 and S5 Figs present total annual precipitation and total annual bare-soil evaporation, respectively, for 2024 and 2025. In addition, panel C of S4 and S5 Figs presents cumulative precipitation and bare-soil evaporation from January to July 2026. We explicitly indicate that these 2026 values represent partial-year accumulations because the year was still in progress at the time of analysis both in the figure captions but also in the Materials and Methods (Lines 306-322). We also refined the macroclimatic values reported in the Results (Lines 462-478) by extracting the corresponding ERA5-Land values directly for the sampling localities, providing a more precise quantitative description of the regional climatic gradient represented in S3–S5 Figs. These additions provide a more consistent climatic context across the three years without altering the interpretation of our results. The new Supporting Information figures are now cited in the main text (Lines 465, 475 and 625).

The study aimed to test the hypothesis that *Atta cephalotes* is more of a habitat specialist, whereas *Atta laevigata* behaves as a more plastic and resilient species. The statistical analysis of niche breadth (defined as the suite of environments and resources across both abiotic and biotic axes that a species can tolerate and utilize) identified four key parameters characterizing the ecology of the two species: canopy openness, association with macro-vegetation types, surface and nest temperatures, and soil pH in the broader area surrounding the main nest entrance.

Initially, I thought that the observed difference of approximately 0.2 pH units between the soils associated with the two species, although statistically significant, was unlikely to be ecologically important. My own expertise lies in humus form classification, and I know that within soil categories inhabited by the same major animal groups, pH can vary by several units. There are, however, threshold values; for example, earthworms generally disappear below pH 4.5 in European soils. After reading the authors’ discussion (lines 466–496), I am persuaded that soil pH, when linked to vegetation changes and their consequences for fungal communities, could indeed influence the ecology of these ants.

Thanks to the Reviewer for this thoughtful comment and are pleased that the Discussion clarified the ecological relevance of the observed soil pH differences despite their relatively small absolute magnitude. We agree that the interpretation should be considered within the broader context of vegetation structure, fungal communities, and the ecology of the ant–fungus symbiosis. No changes to the manuscript were considered necessary.

Overall, the interpretation proposed by the authors is consistent with the analytical results and appears well supported. The manuscript presents an interesting example of how two ant species that coexist at broad spatial scales by exploiting different local microhabitats may become segregated when those microhabitats are incorporated into distinct human-modified environments.

My own work on habitat differentiation has focused on soil microorganisms. These can be sampled relatively easily (a few cubic centimeters of soil) and analyzed at moderate cost; for roughly €100 per sample, one can obtain information on archaeal, fungal, and bacterial communities, both active and inactive. I suspect that microbial communities would differ substantially between the habitats occupied by the two ant species studied here.

We fully agree that characterizing the microbial communities associated with the different habitats occupied by *A. cephalotes* and *A. laevigata* represents a highly promising direction for future research. As the Reviewer points out, differences in archaeal, bacterial, and fungal communities may contribute to the contrasting ecological strategies documented in this study. We have therefore emphasized on the final paragraph of the Discussion that understanding the mechanisms underlying differences in realized niche breadth will require moving beyond the ant alone and considering the broader biological system, including its obligate fungal symbiont. In future work, we also aim to investigate the genomic and transcriptomic responses of both the ants and *Leucoagaricus gongylophorus* across contrasting environmental conditions to better understand the mechanisms underlying differences in realized niche breadth and persistence under land-use change.

I have no further comments. I thank the author for this interesting contribution and look forward to future developments in this area of research.

## **Reviewer 2**

My main conceptual concern is that the ms frames plasticity and specialization as opposing ecological strategies, but this dichotomy is not fully justified. Phenotypic or behavioral plasticity can itself be one of the mechanisms allowing niche specialization, for example by enabling organisms to maintain performance within a narrow set of environmental conditions through flexible behavioral, physiological, or architectural adjustments. Therefore, a narrow niche breadth does not necessarily imply low plasticity. In the current study, niche breadth is inferred from among-colony dispersion in environmental and architectural variables. However, this represents the realized environmental variation occupied by each species, rather than a direct measure of plasticity. Demonstrating plasticity would require evidence of within-species or within-colony responses across environmental gradients, temporal changes, reaction norms, experimental manipulation, or, at minimum, a clearer separation between individual- or population-level plastic responses and species-level habitat associations. For this reason, I suggest that the authors soften claims regarding plasticity unless they provide direct evidence of plastic responses. More importantly, the theoretical framework should be revised so that plasticity is not treated as the opposite of specialization, but rather as a potential mechanism that may either broaden niche breadth or help maintain performance within a specialized niche.

We sincerely thank the Reviewer for this conceptual observation crucial for our entire study. We carefully reconsidered the theoretical framework that we proposed and fully agree that plasticity (either phenotypic or behavioural) and ecological specialization should not be presented as mutually exclusive or opposing strategies. As the Reviewer correctly points out, specialist species may also exhibit plastic behavioural or physiological responses while remaining restricted to a relatively narrow realized niche.

After revising both our hypotheses and the literature, we concluded that the term plasticity did not accurately describe either the questions addressed in this study or the evidence generated by our analyses. Our data quantify differences in **realized niche breadth** across fine-scale environmental gradients, rather than phenotypic or behavioural plasticity.

We moderated our interpretations throughout the Discussion and Conclusions. Rather than presenting realized niche breadth as direct evidence that either species is a generalist or a specialist, we now emphasize that our analyses quantify only the realized niche represented by the environmental variables included in this study. Therefore, we present our conclusions in terms of whether the observed differences in realized niche breadth are **consistent with generalist or specialist** ecological strategies, rather than demonstrating these strategies directly.

Accordingly, we revised the manuscript to replace the **former plasticity versus specialization** framework with a more appropriate **generalist versus specialist** framework, which is better supported by the niche-breadth literature. Throughout the manuscript (e.g. Lines 25, 64–66, 487–490), we now interpret the broader realized niche of *A. cephalotes* as being **consistent** with a more generalist ecological strategy, whereas the narrower realized niche of *A. laevigata* is discussed as being consistent with a more specialist strategy.

We believe these revisions have substantially strengthened the conceptual framework of the manuscript, and we thank the Reviewer for helping us improve this aspect of the study.

## **Reviewer 3**

I found your hypothesis contrasting, require more explanation (i) at the abstract you wrote "two competing hypotheses: first, the Specialist Filter, which posits that A. laevigata exhibits a narrow niche breadth and high fidelity for open grasslands, restricting its success to stable, large-scale pasture matrices; and second, the Plastic Survivor, which predicts that A. cephalotes displays significantly higher environmental (ii) then at the introduction you stated "We tested the contrasting hypotheses of A. cephalotes acting as a habitat specialist and A. laevigata as a plastic survivor."

We sincerely thank the Reviewer for this observation. We agree that the original way we formulated our hypotheses required clearer conceptual justification. After reconsidering the theoretical framework of the study, we concluded that the former terminology of "**plastic survivor versus specialist filter**" did not accurately reflect either our hypotheses or the evidence generated by our analyses. Therefore, we completely revised the conceptual framework throughout the manuscript. We replaced the former plasticity-based terminology with a **generalist versus specialist framework**, which is more appropriate for interpreting differences in realized niche breadth and is better supported by the current ecological literature. The Abstract (Lines 22-25), Introduction (Lines 64-66, 71-79, 135-141), Discussion (Lines 487-490, 495-501), and Conclusions (Line 688) were revised accordingly.

Another points: there are very short about "their potentials predators": you should develop a bit what you wrote in the discussion: something like the potential role of predator in such ecosytem changes regarding these species, for instance give existing example, if there are, about which ones are their natural predators in nature, are they able to survive as well with the ecosystem change based on published information" - if no manuscript exists on that subject, please mentioned it but not only wrote like you did on predator subject. I stress that point as in various taxa, the exposition within land modified expose them to different threars among them are paredators. It might as well new predators" whiar are not their natural ones but can prey on them as they live in open land, etc. So if you have some information on potential ants predators in open land, are we sure they won't prey on them?

We agree with the Reviewer that predation may represent an additional ecological filter influencing the persistence of Atta species in human-modified landscapes. Therefore, we expanded the Discussion (Lines 595–607) to incorporate literature on natural enemies of leaf-cutting ants. These include phorid flies, armadillos, beetles, birds, and spiders. We also discuss the current lack of evidence evaluating whether changes in predator communities across forests, pastures, and urban environments differentially affect *A. cephalotes* and *A. laevigata*. Furthermore, we included the traditional harvest of reproductive females of A. laevigata for human consumption in the Santander region as an additional anthropogenic pressure that may deserve future investigation. Because neither predation nor human harvesting was quantified in the present study, we deliberately discuss these processes as potential ecological filters and future research directions rather than as mechanisms explaining our observed patterns.
